# Single-point *ACT2* gene mutation in the *Arabidopsis* root hair mutant *der1-3* affects overall actin organization, root growth and plant development

**DOI:** 10.1093/aob/mcx180

**Published:** 2017-12-27

**Authors:** L Vaškebová, J Šamaj, M Ovečka

**Affiliations:** Department of Cell Biology, Centre of the Region Haná for Biotechnological and Agricultural Research, Palacký University Olomouc, Olomouc, Czech Republic

**Keywords:** *ACTIN2* gene, actin cytoskeleton, *Arabidopsis thaliana*, cell division plane orientation, *der1-3* mutant, GFP-FABD2, live-cell imaging, microscopy, microtubules, phenotype, plant development, root growth

## Abstract

**Background and Aims:**

The actin cytoskeleton forms a dynamic network in plant cells. A single-point mutation in the *DER1* (*deformed root hairs1*) locus located in the sequence of *ACTIN2*, a gene for major actin in vegetative tissues of *Arabidopsis thaliana*, leads to impaired root hair development (Ringli C, Baumberger N, Diet A, Frey B, Keller B. 2002. ACTIN2 is essential for bulge site selection and tip growth during root hair development of *Arabidopsis*. *Plant Physiology***129:** 1464–1472). Only root hair phenotypes have been described so far in *der1* mutants, but here we demonstrate obvious aberrations in the organization of the actin cytoskeleton and overall plant development.

**Methods:**

Organization of the actin cytoskeleton in epidermal cells of cotyledons, hypocotyls and roots was studied qualitatively and quantitatively by live-cell imaging of transgenic lines carrying the GFP-FABD2 fusion protein and in fixed cells after phalloidin labelling. Patterns of root growth were characterized by FM4-64 vital staining, light-sheet microscopy imaging and microtubule immunolabelling. Plant phenotyping included analyses of germination, root growth and plant biomass.

**Key Results:**

Speed of germination, plant fresh weight and total leaf area were significantly reduced in the *der1-3* mutant in comparison with the C24 wild-type. Actin filaments in root, hypocotyl and cotyledon epidermal cells of the *der1-3* mutant were shorter, thinner and arranged in more random orientations, while actin bundles were shorter and had altered orientations. The wavy pattern of root growth in *der1-3* mutant was connected with higher frequencies of shifted cell division planes (CDPs) in root cells, which was consistent with the shifted positioning of microtubule-based preprophase bands and phragmoplasts. The organization of cortical microtubules in the root cells of the *der1-3* mutant, however, was not altered.

**Conclusions:**

Root growth rate of the *der1-3* mutant is not reduced, but changes in the actin cytoskeleton organization can induce a wavy root growth pattern through deregulation of CDP orientation. The results suggest that the *der1-3* mutation in the *ACT2* gene does not influence solely root hair formation process, but also has more general effects on the actin cytoskeleton, plant growth and development.

## INTRODUCTION

The plant cytoskeleton, consisting of actin filaments (AFs) and microtubules, represents a dynamic supramolecular structure with many cellular functions. The actin cytoskeleton plays crucial roles in the establishment of cell polarity, in the positional control and progression of cell division, and it is involved in diffuse and polar cell elongation (Volkmann and Baluška, 1999; Baluška *et al.*, 2000b; Barlow and Baluška, 2000; Meagher and Fechheimer, 2003; Šamaj *et al.*, 2004; Henty-Ridilla *et al.*, 2013). The genome of *Arabidopsis thaliana* contains two major classes of actin genes, encoding vegetative and reproductive actin isoforms. The vegetative group of actin genes includes *ACT2*, *ACT7* and *ACT8*, which are typically expressed in all vegetative tissues. The reproductive class of actin genes consists of *ACT1*, *ACT3*, *ACT4*, *ACT11* and *ACT12*. They are expressed mainly in pollen tubes and ovules (Meagher *et al.*, 1999; Kandasamy *et al.*, 2007). Vegetative actin isoforms have specific expression patterns. The *ACT2* gene is expressed in young and old vegetative tissues, in flowers, leaves, stems and roots. The *ACT8* gene has expression patterns similar to those of the *ACT2* gene while the *ACT7* gene is expressed mainly in young expanding vegetative tissues (Meagher *et al.*, 1999). Importantly, the overproduction of ACT1 caused the formation of sheet- or star-like aberrant actin structures in vegetative cells, leading to different thickness and orientation of actin filaments in comparison with control cells (Kandasamy *et al.*, 2002). On the other hand, overexpression of *ACT2* in vegetative tissues has little effect on plant morphology and the structure of actin filaments (Kandasamy *et al.*, 2002). Although the vegetative actins differ from the reproductive ones only by 4–7 % at the amino acid sequence level, their expression patterns and functions are different (Kandasamy *et al.*, 2007).

Some vegetative plant organs contain up to 50 % of *ACT2* mRNA out of the total actin mRNA amount, suggesting that the *ACT2* gene is highly expressed among actin genes in *Arabidopsis* (McDowell *et al.*, 1996). An expression study of *proACT2::GUS* and *proACT8::GUS* constructs revealed that the *ACT2* promoter is the stronger (An *et al.*, 1996). Its expression was found in nearly all vegetative tissues of young seedlings and juvenile and mature plants, and persisted in older plant tissues. No or little expression has been found in the seed coat, hypocotyl, gynoecium or pollen sac (An *et al.*, 1996). Hence, the ACT2 isoform has attracted more interest than other isoforms in plant biology studies. Mutational studies of the *ACT2* gene generated several interesting mutants. Classical mutagenesis approaches based on ethyl methanesulphonate (EMS) or X-rays, inducing randomly generated single-point mutations, led to the isolation of a series of *der* mutants (*der1-der9*; *deformed root hair*) (Ringli *et al.*, 2002, 2005). Three *der1* allelic mutants isolated after EMS mutagenesis in the C24 ecotype background were identified based on the root hair phenotype. Among them, the strongest phenotypic effect has been described in *der1-2* and *der1-3* mutants (Ringli *et al.*, 2002). The root hair phenotype of the *der1-3* mutant was characterized by wrong selection of the root hair initiation site in the trichoblast and impairment of root hair elongation after bulge establishment. Therefore, *der1-3* mutant plants show a phenotype of very short root hairs (Ringli *et al.*, 2002). It has been concluded that ACT2 is involved mainly in the tip growth of root hairs, since no other obvious aberrations of overall development and phenotype of *der1* mutant plants have been detected (Ringli *et al.*, 2002). Another mutant produced by EMS mutagenesis in the *Ler* ecotype background, *act2-2D*, is not able to form root hairs. In this mutant, bulges are not developed on trichoblasts (root hair-forming cells) and bundles of actin filaments in the root epidermal cells of *act2-2D* mutant are shorter than in wild-type plants (Nishimura *et al.*, 2003). T-DNA insertional loss-of-function mutants in *ACT2*, *act2-1* (Gilliland *et al.*, 2002) and *act2-3* (Nishimura *et al.*, 2003) show very similar root hair phenotypes. The length of root hairs of the *act2-1* mutant was 10–70 % of that of wild-type root hairs (Gilliland *et al.*, 2002). Root hair initiation is not impaired in the *act2-3* mutant, but mature root hairs of this mutant are shorter than wild-type root hairs (Nishimura *et al.*, 2003). These data support the role of AFs in the process of root hair initiation and in their tip growth (Braun *et al.*, 1999; Baluška *et al.*, 2000a; Pei *et al.*, 2012). The phenotype of the *act2-5* mutant is a bit more complex, showing a wavy shape of the main root and altered structure of the actin cytoskeleton (Lanza *et al.*, 2012). Single mutants for the vegetative actin genes *ACT2*, *ACT7* and *ACT8* revealed only mild phenotypes, but double mutants were much more affected. They exhibited dwarf phenotypes, defects in cell and organ morphology and aberrant actin cytoskeleton organization (in *act2-1 act7-4* and *act8-2 act7-4*) or totally missing root hairs (*act2-1 act8-2*) (Kandasamy *et al.*, 2009). This study revealed that ACT7 is involved in root growth, epidermal cell specification, cell division and root architecture, while ACT2 and ACT8 are essential for root hair tip growth. Nevertheless, all mutants for single vegetative actin genes were fully fertile (Gilliland *et al.*, 1998).

The plant cytoskeleton plays important roles during cell division, a fundamental process for plant morphogenesis and development. Plant cells are delineated by rigid cell walls and therefore the orientation of the cell division plane (CDP) during cell division is a basic determinant of plant anatomy and development (Müller, 2012; Smolarkiewicz and Dhonukshe, 2013; Zhang *et al.*, 2016). Plant cell division is controlled by specific arrangements of mitotic microtubules organized in distinct arrays. The microtubule preprophase band (PPB), formed at preprophase and early prophase, predicts the future cell division site. The mitotic spindle is a major mitotic microtubule structure formed at prophase, which positions and segregates sister chromatids through the successive stages of metaphase, anaphase and telophase. The phragmoplast is a specialized microtubule array formed during cytokinesis, delivering vesicles to the nascent cell plate and expanding centrifugally to meet the cortical domain predetermined by the PPB (Fowler and Quatrano, 1997; Rasmussen *et al.*, 2013; de Keijzer *et al.*, 2014). The indispensable role of microtubules in the determination of CDP orientation of dividing cells has been proved in many studies after pharmacological treatments or in appropriate mutants (Palevitz, 1987; Mineyuki *et al.*, 1991; Komis *et al.*, 2017; reviewed in Rasmussen *et al.*, 2013).

The actin cytoskeleton also contributes to the correct progression of cell division, although AFs differ from microtubules in spatial and temporal distribution. Actin filaments contribute to early stages of PPB formation, but are less abundant in the PPB at later stages (Takeuchi *et al.*, 2016), while their occurrence in the mitotic spindle is obscure, forming a surrounding cage around the spindle to maintain its position during mitosis (Lloyd and Traas, 1988; Katsuta *et al.*, 1990). Later, AFs are abundant in the phragmoplast during cytokinesis. Not surprisingly, disorientation of the CDP in tobacco BY-2 cells has been induced after application of actin-depolymerizing or actin-stabilizing drugs during early stages of mitosis (Sano *et al.*, 2005; Kojo *et al.*, 2013, 2014). These data suggest that both microtubules and AFs may functionally cooperate during PPB and phragmoplast formation, the establishment of the CDP and the correct deposition of the cell plate. Reciprocity between microtubule and AF organization was shown through the partial damage of the fine transversely oriented cortical AFs after microtubule depolymerization in *A. thaliana* and carrot interphase cells. Vice versa, pharmacological disruption of AFs led to the reorganization of microtubules (Sampathkumar *et al.*, 2011). These data strongly indicate that AFs may play a key role not only in the assembly but also in the positioning of the PPB in the cell. It is not clear, however, how the process of microtubule–AF interaction during the regulation of cell division is affected in *ACTIN* mutants.

So far, only the root hair phenotype has been described in *der1* mutants without any obvious aberrations in plant development. In the present study, we provide thorough plant phenotyping and characterization of the actin cytoskeleton in *der1-3* mutant plants. The changed organization and arrangement of AFs in cell types other than root hairs, the phenotypical differences in root development related to the deregulated CDP during cell division and the changed leaf phenotype indicate that the *der1-3* mutation in the *ACT2* gene has effects additional to those on root hair formation.

## MATERIALS AND METHODS

### Plant material and growth conditions

Seeds of *Arabidopsis thaliana* (L.) Heynh. ecotype C24 and the *der1-3* (*deformed root hairs1*) mutant (Ringli *et al.*, 2002) were surface-sterilized and placed on half-strength Murashige and Skoog (MS) medium without vitamins solidified with 0.6 % w/v Phytagel. Petri dishes containing seeds were stratified at 4 °C for 3 d. After stratification, seeds were cultivated vertically in a growth chamber at 21 °C at 70 % humidity with a 16/8 h light/dark photoperiod (*in vitro* conditions). For experiments *in vivo*, seeds were imbibed overnight in sterile tap water at room temperature. Seeds were transferred to small flowerpots with soil containing Careo (pesticide). Plants were cultured in a growth chamber at 24 °C at 60 % humidity with a 16/8 h light/dark photoperiod.

### Stable plant transformation

Stable plant transformation was performed according to Clough and Bent (1998). *Arabidopsis thaliana* (ecotype C24 and der1-3 mutant) plants were transformed with *Agrobacterium tumefaciens* strain GV3101 carrying a construct *pro35S::GFP:FABD2*, coding for F-actin binding domain 2 of *Arabidopsis* FIMBRIN 1 (FABD2) fused to green fluorescent protein (GFP; Voigt *et al.*, 2005) or with a construct *pro35S::GFP:MBD* coding for the microtubule-binding domain (MBD) of the mammalian MICROTUBULE-ASSOCIATED PROTEIN 4 (MAP4) fused to GFP (Marc *et al.*, 1998). Both constructs were driven by the constitutive 35S promoter. An N-terminal GFP fusion with rifampicin and kanamycin resistance was prepared by a classical cloning method in pCB302 vector with the herbicide phosphinothricin as the selection marker *in planta*. Seeds of the *T*_1_ generation were plated on the selection culture medium with phosphinothricin (50 mg mL^−1^). The root hair phenotype of transgenic *der1-3* plants was visually selected (using a stereomicroscope) and the presence of marker GFP fusion proteins was confirmed using a fluorescence microscope. Seeds of the *T*_3_ generation were used for experiments.

### FM4-64 and phalloidin staining

The FM4-64 dye was used as a plasma membrane marker of root cells. Three-day-old seedlings of *A. thaliana* ecotype C24 and *der1-3* mutant were placed in a drop of half-strength MS culture medium supplemented with 4 μm FM4-64 (Invitrogen) on a microscope slide for 30 min in darkness and at constant humidity. After staining, excess FM4-64 was carefully washed from the slide using culture medium, and samples were then directly observed in a spinning-disk microscope.

Visualization of F-actin using phalloidin was performed in 3-d-old plants of *A. thaliana* ecotype C24 and *der1-3* mutant according to Panteris *et al.* (2006). After fixation, F-actin was labelled with Alexa Fluor 488–phalloidin (Invitrogen). Samples were observed with a confocal laser scanning microscope.

### Immunolabelling of microtubules using a whole-mount method

Three-day-old seedlings of C24 and *der1-3* mutant cultivated on half-strength MS medium were prepared for whole-mount immunofluorescence labelling of roots according to a standard protocol (Beck *et al.*, 2010; Šamajová *et al.*, 2014) with some modifications. Samples were fixed at room temperature for 1 h or at 4 °C overnight. Washing of samples after aldehyde reduction was done five times for 10 min and washing after cell wall digestion was done three times for 10 min. For blocking we used 5 % w/v bovine serum albumin (BSA) in phosphate-buffered saline (PBS). As the primary anti-α tubulin rat monoclonal antibody we used clone YOL1/34 (Bio-Rad) diluted in 3 % w/v BSA, and thorough washing after incubating with the primary antibody was done 12 times for 10 min with 3 % w/v BSA in PBS. Subsequently, a secondary Alexa Fluor 488-conjugated anti-rat IgG antibody (Molecular Probes), appropriately diluted in 3 % w/v BSA, was applied and incubation was done at 37 °C for 3 h followed by incubation at 4 °C overnight. Nuclear DNA was counterstained with 4′,6-diamidino-2-phenylindole (DAPI; Sigma Aldrich). Samples were examined with a confocal laser scanning microscope.

### Microscopy

#### Confocal laser scanning microscopy

Fixed plant samples for phalloidin staining and for whole-mount immunolabelling were examined with a confocal laser scanning microscope (LSM 710; Carl Zeiss, Germany) equipped with a Plan-Apochromat ×40/1.4 numerical aperture (NA) oil immersion objective. Samples labelled with phalloidin were imaged using a 488-nm laser excitation line and emission spectrum 493–630 nm. Laser excitation intensity did not exceed 2 % of the laser intensity range available. The range of the Z-stack was always set to 0.61 µm. Immunolabelled roots were imaged using a 488-nm excitation laser line and emission spectrum 493–630 nm for Alexa Fluor 488 fluorescence detection and with a 405-nm excitation laser line and emission spectrum 410–495 nm for DAPI fluorescence detection.

#### Spinning disk microscopy

Live-cell imaging of the cytoskeleton and FM4-64-labelled samples was performed with a spinning-disk microscope (Cell Observer Z.1; Carl Zeiss, Germany), equipped with an EC Plan-Neofluar ×40/1.3 NA oil immersion objective and Plan-Apochromat ×63/1.4 NA oil immersion objective. Samples were imaged with a 488-nm excitation laser line and BP525/50 emission filter for detection of GFP fluorescence and emission filter BP690/50 for detection of FM4-64 fluorescence. Living samples expressing *pro35S::GFP:FABD2* and *pro35S::GFP:MBD* constructs were scanned every 30 s for 30 min. Images were recorded with a high-resolution Evolve 512 back-thinned EM-CCD camera (Photometrics) with the exposure time 500–750 ms per optical section.

#### Light-sheet microscopy

Live-cell imaging of whole roots was done with a light-sheet fluorescence microscope (Lightsheet Z.1; Carl Zeiss, Germany) equipped with a W Plan-Apochromat ×20/1.0 NA water immersion detection objective and two LSFM ×10/0.2 NA illumination objectives. Samples were imaged using dual-side illumination with a light sheet modulated in pivot scan mode, with a 488-nm excitation line and BP505-545 emission filter. Laser excitation intensity did not exceed 3 % of the laser intensity range available. Image acquisition was done every 5 min in Z-stack mode for 10–15 h. Scaling of images in the *x*, *y* and *z* dimensions was 0.228 × 0.228 × 0.477 µm. Images were recorded with a PCO.Edge camera (PCO AG) with an exposure time of 50 ms per optical section. Samples were prepared in an open system according to Ovečka *et al.* (2015). Sterilized seeds of C24 and the *der1-3* mutant expressing *pro35S::GFP:FABD2* construct were plated on half-strength MS medium and stratified at 4 °C for 5 d. After rupture of the testa, seeds were transferred to culture medium on Petri plates and left to germinate horizontally in a growth chamber. After germination, a piece of fluorinated ethylene propylene (FEP) tube with an inner diameter of 2.8 mm and wall thickness of 0.2 mm (Wolf-Technik, Germany) was inserted in the medium, enclosing the seedling. The FEP tube, filled with a cylinder of solidified culture medium and one seedling, was carefully pulled away from the Petri dish. Roots of the examined plant grew inside the culture medium and leaves were in open space and in contact with the air. The FEP tube was then attached to a glass capillary with inner diameter 2.5 mm serving as a sample holder and placed in the light-sheet microscope.

### Measurements and quantitative and statistical analyses

Plants cultured *in vitro* were scanned directly on Petri plates every 24 h for 5 d from the day of germination. Pictures from a scanner (Image Scanner III, EpsonScan) were used for measurements of primary root length. Phenotypes of 18-d-old plants *in vitro*, rosettes of 19-d-old plants growing *in vivo* and the sizes of individual leaves were documented using a Nikon 7000 camera equipped with macro-objective (50 mm, 2.8; Sigma). The phenotypes of entire roots and root tips of 5-d-old plants *in vitro* were recorded with an M165FC stereo microscope equipped with LAS V 4.0 software (Leica). Microscopic data were processed and evaluated in Zen 2014 software, black and blue editions (Carl Zeiss, Germany). Root growth, orientation of the CDP and cytoskeletal skewness (a measure of microtubule bundling) parameters were measured in ImageJ software. Quantification of CDP orientation was performed from roots of C24 wild-type and *der1-3* mutant plants after FM4-64 staining. The angular positioning of cross-walls with respect to the root axis was measured on microscopic images of root cells labelled with FM4-64. The data obtained were divided into three categories according to the recorded angles: cross-walls at right angles (90° ± 5 %), cross-walls at acute angles (<85°) and cross-walls with obtuse angles (>95°). Angles were measured in root epidermal and cortical cell files of C24 wild-type and *der1-3* mutant plants. Graphs depicting relative frequencies of angle distributions were produced in Microsoft Excel software. Quantitative analysis of actin filament angular distribution was done with CytoSpectre Version 1.2 software using the cell axis as a reference (Kartasalo *et al.*, 2015). Analysis showed the degree of actin filament arrangement with respect to transverse (defined as perpendicular to the cell axis at an angle of 0° or 180°), longitudinal (parallel to the cell axis at an angle of 90° or 270°) or random (an angle between 0° and 180°) orientations. All graphical plots were prepared in Microsoft Excel software and statistical analyses were done using STATISTICA 12 (StatSoft) software using analysis of variance (ANOVA) and subsequent Fisher’s LSD tests (*P* < 0.05).

## RESULTS

Phenotype, growth and biomass production of *der1-3* mutant plants

**Fig. 1.**
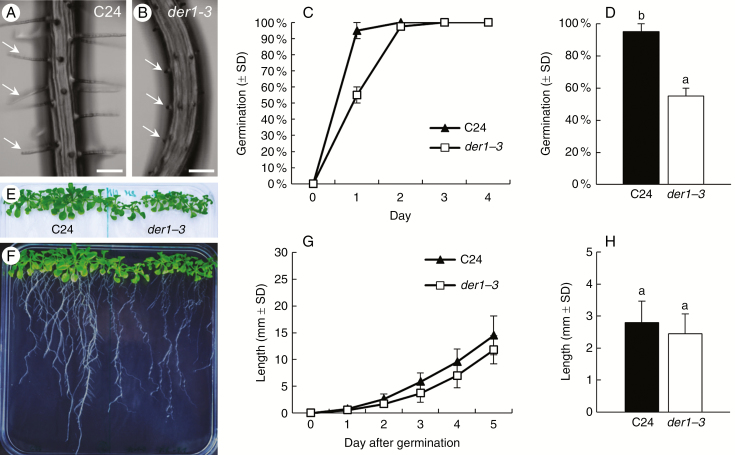
Germination, root growth and phenotype overview of *der1-3* mutant. (A, B) Mature part of the root of 5-d-old seedlings showing normal long root hairs in C24 wild-type (A, arrows) and much shorter root hairs arrested at the bulge stage in *der1-3* mutant (B, arrows). (C, D) Germination of C24 and *der1-3* seeds. Efficiency of seed germination within first 4 d after placing seeds on Phytagel-solidified culture medium (C) and average germination rate within the first 24 h (D). Experiments were performed twice with 20 seeds per line. (E, F) Size and phenotype of green aerial parts (E) and entire 18-d-old plants (F) grown on Phytagel-solidified culture medium *in vitro*. (G, H). Time course of root growth of plants cultured on Phytagel-solidified culture medium *in vitro* within the first 5 d after germination (G) and average root growth per 24 h (H). Experiments were performed twice with eight plants per line. Different letters above bars represent statistical significance at *P* < 0.05 (one-way ANOVA). Scale bars (A, B) = 100 µm.

A single-point mutation in the *DER1* (*deformed root hairs1*) locus within the *ACTIN2* gene in *A. thaliana* produced by EMS-based mutagenesis leads to a mutant phenotype with impaired root hair development (Ringli *et al.*, 2002). The process of root hair formation is affected after root hair initiation and root hair tip growth. Among three allelic mutations originally identified, *der1-3* shows the strongest phenotype (Ringli *et al.*, 2002). Direct comparison revealed that root hairs of the *der1-3* mutant were much shorter than in the C24 wild-type (Fig. 1A, B). They showed arrested development mainly after bulge formation, confirming the root hair phenotype of this mutant described earlier (Ringli *et al.*, 2002, 2005).

Further morphological analysis evaluated potential differences in the phenotype, growth and development of whole roots and entire plants between control C24 wild-type and *der1-3* mutant. Our phenotypic studies comprised seed germination tests, evaluation of the root growth rate of young seedlings and biomass quantification in more advanced plants under *in vitro* and *in vivo* conditions. Survival and progress of early plant growth and development are highly dependent on the efficiency and speed of seed germination. For this reason we analysed germination rates of seeds that were surface-sterilized at a dry stage, thoroughly washed, primed at 4 °C for 2 d and aseptically cultured on Phytagel-solidified medium in a vertical position under controlled conditions. Importantly, the germination rate of *der1-3* mutant seeds was considerably slower compared with wild-type (Fig. 1C). Although all seeds were treated similarly and the germination efficiency was eventually 100 %, seeds of the *der1-3* mutant germinated significantly later in comparison with control C24 wild-type seeds. Within the first 24 h after placing of seeds in the growing chamber, 95 ± 0.05 % of the C24 wild-type seeds germinated, while only 55 ± 0.05 % of *der1-3* mutant seeds started germination in same time period (Fig. 1D).

Size comparison of 18-d-old entire plants revealed visually smaller green aerial parts (Fig. 1E, F) and a less developed root system (Fig. 1F) of *der1* mutants. However, measurement of root growth rates within the first 5 d after germination showed no significant difference between C24 wild-type and *der1-3* mutant plants (Fig. 1G). Similar results were found in a comparison of average root growth per 24 h measured within the first 5 d after germination, with no significant difference between C24 wild-type and *der1-3* mutant plants (Fig. 1H). These data indicate that the visually shorter roots of *der1-3* mutant plants are attributable to delays in seed germination (Fig. 1C, D) rather than to a slower root growth rate (Fig. 1G, H).

**Fig. 2.**
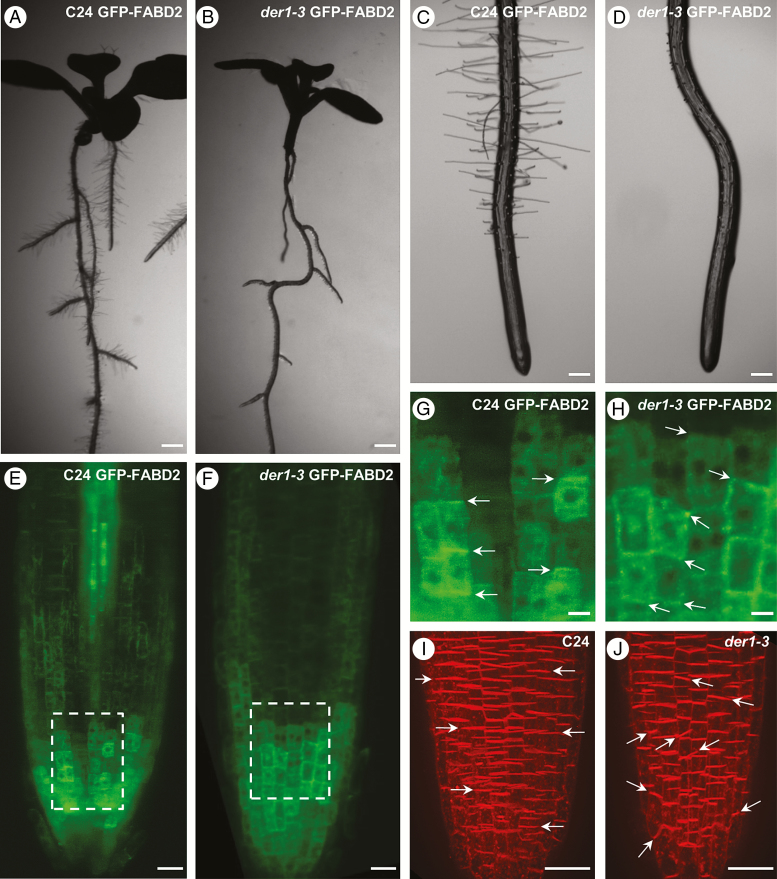
Root phenotype and pattern of root growth in *der1-3* mutant. (A, B) Root morphology of 8-d-old seedlings of C24 wild-type (A) and *der1-3* mutant (B) grown on Phytagel-solidified culture medium *in vitro*. (C, D) Root tip morphology of 5-d-old seedlings documenting straight shape of the root in C24 wild-type (C) and wavy shape of the root in *der1-3* mutant (D). (E–H) Light-sheet fluorescence microscopy imaging of transgenic *Arabidopsis* lines harbouring the actin filament fluorescent marker GFP-FABD2. (E, F) Overview of GFP-FABD2 expression in root apex cells of C24 wild-type (E) and *der1-3* mutant (F). (G, H) Detailed images of cell files in the root apex of C24 (G, magnified inset from E) and *der1-3* mutant (H, magnified inset from F) harbouring the GFP-FABD2 marker. Cell boundaries within the cell files at cross-walls defining orientation of cell division planes during cell division are marked by arrows. (I, J) Vital labelling of root apices using the fluorescent membrane marker FM4-64. There is prominent FM4-64 labelling of plasma membranes at the cross-walls of C24 wild-type (I) and *der1-3* mutant (J) root cells. Arrows indicate orientation of cell division planes during cell division. Scale bars (A, B) = 400 µm; (C, D) = 200 µm; (E, F, I, J) = 20 µm; (G, H) = 5 µm.

Nevertheless, the pattern of root growth in the *der1-3* mutant displayed some alterations, leading to changes in phenotype of the whole root system. In comparison with C24 wild-type, where primary roots developed in a straight direction along a longitudinal vector (Fig. 2A, C), primary roots of *der1-3* mutant showed a more irregular and wavy growth pattern (Fig. 2B, D). A similar trend was observed in lateral roots of the *der1-3* mutant (Fig. 2B), which are normally straight in the C24 wild-type (Fig. 2A). To decipher the role of actin in root waving during growth, we analysed AF organization in the apex of primary roots during the root growth of C24 and *der1-3* seedlings stably expressing a *35S::GFP-FABD2* actin marker. In order to maintain intact living plants in near-environmental conditions and avoid any artefacts in the speed and directionality of root growth induced by prolonged irradiation during imaging, we utilized light-sheet microscopy. This method provides an optimal approach for long-term developmental microscopy imaging of intact plants with minimal fluorophore photobleaching and phototoxicity. The actin cytoskeleton was visualized in cells of the whole root apex of transgenic C24 (Fig. 2E) and *der1-3* mutant (Fig. 2F) plants. Detailed images of the root apex revealed boundaries between individual cells at their cross-walls in cell files. The planes of these cross-walls in non-dividing cells corresponded to CDP orientation during cell division. Cross-walls between individual cells were predominantly transverse in cell files of C24 control (Fig. 2E, G). In contrast, many cross-walls between individual cells were obliquely oriented in cell files of *der1-3* mutant root (Fig. 2F, H). In order to quantify cross-wall orientation differences between C24 and *der1-3* seedlings, roots were stained with FM4-64 and examined by spinning-disk fluorescence microscopy. Inspection of C24 wild-type roots confirmed the preferential perpendicular orientation of the cross-walls of cell files with respect to the longitudinal root axis (Fig. 2I), and the frequent presence of oblique orientation of the cross-walls in cell files of *der1-3* mutant roots (Fig. 2J).

**Fig. 3.**
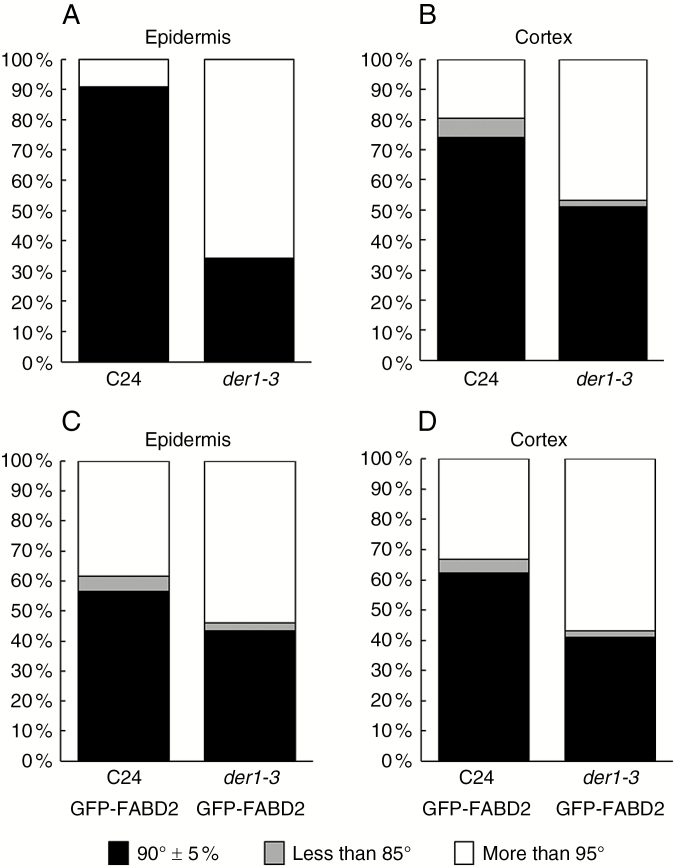
Quantitative evaluation of cross-wall orientation between neighbouring cells in root cell files, indicating orientation of cell division planes. Data were collected from roots labelled with FM4-64. Angles between the cross cell wall and the longitudinal cell wall of measured cells were scored into three categories: right angle (90° ± 5 %); acute angle (<85°); and obtuse angle (>95°). (A, B) Relative frequencies of angle distributions in epidermal and cortical cell files of C24 wild-type and *der1-3* mutant plants. (C, D) Relative frequencies of angle distributions in epidermal and cortical cell files of transgenic C24 and *der1-3* plants harbouring the actin filament fluorescent marker GFP-FABD2. Experiments were performed three times with 10–17 cells from three plants per line.

Quantitative evaluation of cross wall orientations between neighbouring cells in cell files was performed in FM4-64-labelled roots by measuring the angle between the cross and the longitudinal wall of the same cell. CDP orientations of measured cells were scored into three categories: close to right angle (90° ± 5 %), with acute angles (<85°) and with obtuse angles (>95°). Data were collected from cells in cell files of root epidermis and cortex. To exclude the possible influence of GFP-FABD2 overexpression on CDP orientation, the same measurements were done in both transgenic and non-transformed C24 and *der1-3* seedlings. Such measurements confirmed the prevalence of transverse CDP orientation in the epidermis and the cortex of C24 wild-type roots (Fig. 3A, B). In contrast, a significantly lower number of perpendicular CDP orientations was present in the epidermis and cortex of *der1-3* mutant roots, while oblique CDP orientations increased (Fig. 3A, B). A proportionally lower number of perpendicular CDP orientations was found in epidermal and cortical cell files of transgenic C24 and *der1-3* mutant lines harbouring GFP-FABD2 compared with control non-transgenic C24 wild-type and *der1-3* mutant lines (Fig. 3C, D). Nevertheless, a significantly lower number of perpendicular CDP orientations was present in *der1-3* GFP-FABD2 mutant roots, while the number of oblique CDP orientations was increased in both cell layers (Fig. 3C, D). These results clearly indicate that the wavy pattern of root growth in the *der1-3* mutant is somehow related to alterations in CDP orientations during cell division in the root meristem.

**Fig. 4.**
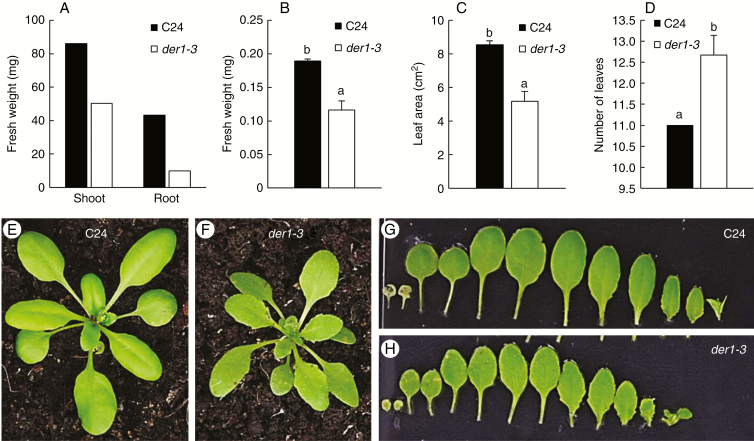
Development and phenotype of shoots in *der1-3* mutant. (A) Average fresh weight of shoots and roots of 19-d-old C24 wild-type and *der1-3* mutant plants grown on Phytagel-solidified culture medium *in vitro*. Shoots and roots were collected separately from ten plants and weighed together. The experiment was performed three times. (B–D) Quantitative parameters of shoots of 19-d-old C24 wild-type and *der1-3* mutant plants grown in pots (*in vivo*): fresh weight of green aerial plant parts (B), total area of true leaves per plant (C) and number of true leaves per plant (D). Data were collected from three plants. (E–H) Comparison of leaf rosettes and individual true leaves in 19-d-old plants grown in pots (*in vivo*). Size of the leaf rosette in C24 wild-type plants (E) and *der1-3* mutant plants (F). Number and size of individual true leaves in C24 wild-type plants (G) and *der1-3* mutant plants (H). Different letters above bars represent statistical significance at *P* < 0.05 (one-way ANOVA).

To reveal whether the development of green aerial parts (Fig. 1E, F) could also be significantly affected by delay in seed germination in the *der1-3* mutant, we compared quantitative parameters of shoots and leaves. Shoot and root fresh weights of 19-d-old plants cultured *in vitro* were significantly higher in the control C24 wild-type plants than in the *der1-3* mutant (Fig. 4A). Nevertheless, average fresh weight of all true leaves (Fig. 4B), average leaf area (Fig. 4C), size of leaf rosettes (Fig. 4E, F) and size of individual leaves (Fig. 4G, H) in plants cultured *in vivo* suggested that the reduced development of the *der1-3* mutant might not be related to delay in germination. This suggestion was supported by the higher average number of leaves in 19-d-old *der1-3* mutant plants (Fig. 4D), which may functionally compensate for the suppressed development of green aerial part in *der1-3* mutants.

### Organization of the actin cytoskeleton

We performed detailed qualitative and quantitative analyses of the actin cytoskeleton in the *der1-3* mutant in order to see how the *ACT2* mutation could be involved in the phenotypical and developmental changes of this mutant. The AFs were visualized in hypocotyls and cotyledons of fixed and fluorescent phalloidin-labelled 3-d-old seedlings. This was followed by live-cell imaging in hypocotyl and root epidermal cells of transgenic lines expressing the GFP-FABD2 marker for AFs. These microscopic observations were supported by quantitative analysis of AF angular distribution in transverse (defined as perpendicular to the cell axis at an angle of 0° or 180°), longitudinal (parallel to the cell axis at an angle of 90° or 270°) or random (angles between 0° and 180°) orientations (Kartasalo *et al.*, 2015).

**Fig. 5.**
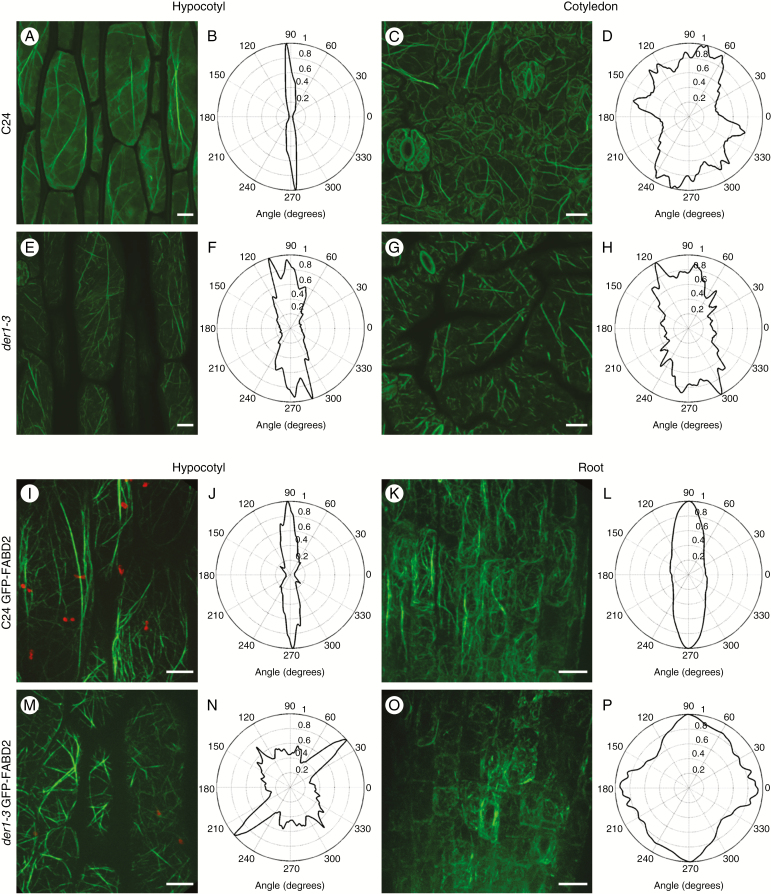
Organization of the actin cytoskeleton in C24 wild-type and *der1-3* mutant plants. (A–H) Microscopic observations (A, C, E, G) and quantitative analysis (B, D, F, H) of actin filament angular distribution in hypocotyl epidermal cells (A, B, E, F) and cotyledon epidermal cells (C, D, G, H) of 3-d-old C24 wild-type and *der1-3* mutant plants after Alexa Fluor 488–phalloidin labelling of actin filaments. (I–P) Microscopic observations (I, K, M, O) and quantitative analysis of actin filament angular distribution (J, L, N, P) in hypocotyl epidermal cells (I, J, M, N) and in root epidermal cells (K, L, O, P) of 3-d-old C24 wild-type and *der1-3* mutant transgenic plants harbouring the actin filament fluorescent marker GFP-FABD2. Quantitative analysis of actin filament angular distribution was performed for three independent microscopic images of examined cell types from three to five plants. Scale bars = 10 µm.

Actin visualization using Alexa Fluor 488–phalloidin labelling in hypocotyl epidermal cells revealed the presence of long AFs and actin bundles, arranged mostly in longitudinal or slightly oblique orientations in C24 plants (Fig. 5A). Quantification of angular distribution confirmed a rather uniform longitudinal orientation of AFs in these cells (Fig. 5B). In cotyledon epidermal cells of C24 plants AFs were arranged in long, interconnected and curled cables (Fig. 5C), longitudinally and randomly spanning whole cells (Fig. 5D). In contrast, hypocotyl epidermal cells of the *der1-3* mutant contained thinner and shorter AFs and actin bundles (Fig. 5E), which were less pronounced and reorganized in comparison with the C24 control plants (Fig. 5A). In addition, the angular distribution of AFs in the mutant revealed more random AF orientation compared with the C24 control plants (Fig. 5F). Cotyledon epidermal cells of the *der1-3* mutant contained predominantly fragmented AFs and short actin bundles (Fig. 5G), arranged loosely along the longitudinal axes of these cells (Fig. 5H).

Live-cell imaging of the actin cytoskeleton in epidermal hypocotyl cells of transgenic C24 lines carrying the GFP-FABD2 molecular marker revealed actin bundles spanning these cells longitudinally as well as longitudinally, and obliquely oriented fine AFs forming a dense meshwork (Fig. 5I). Thus, quantification of angular distribution revealed a high degree of longitudinal orientation of AFs (Fig. 5J). Organization of the actin cytoskeleton in post-mitotic elongating cells of root epidermis showed typical AFs and actin bundles interconnected into a dense network. Long actin cables formed typical bending structures in the vicinity of cross-walls (Fig. 5K). The angularity of such an arrangement was thus characterized by regular distribution of AFs with prevalent longitudinal orientation (Fig. 5L). The actin cytoskeleton in hypocotyl epidermal cells of the *der1-3* mutant carrying the GFP-FABD2 molecular marker consisted of very short AFs arranged in a random and loose network. In addition, thick and rather short actin cables were oriented preferentially in oblique orientation with respect to the longitudinal axis of the hypocotyl epidermal cells (Fig. 5M). Quantification of AF angularity confirmed random and oblique orientation of AFs (Fig. 5N). The actin cytoskeleton in post-mitotic elongating root epidermal cells of the *der1-3* mutant carrying the GFP-FABD2 molecular marker showed dense arrangements of very short filaments and spot-like structures. The network of AFs was disorganized and actin cables were short and sometimes fragmented (Fig. 5O). Quantitative evaluation of angularity clearly revealed the random orientation of the actin cytoskeleton (Fig. 5P). These data conclusively confirmed marked alterations in the organization of the actin cytoskeleton in cells of different tissues and organs of *der1-3* mutant plants caused by mutation of *ACT2*.

**Fig. 6.**
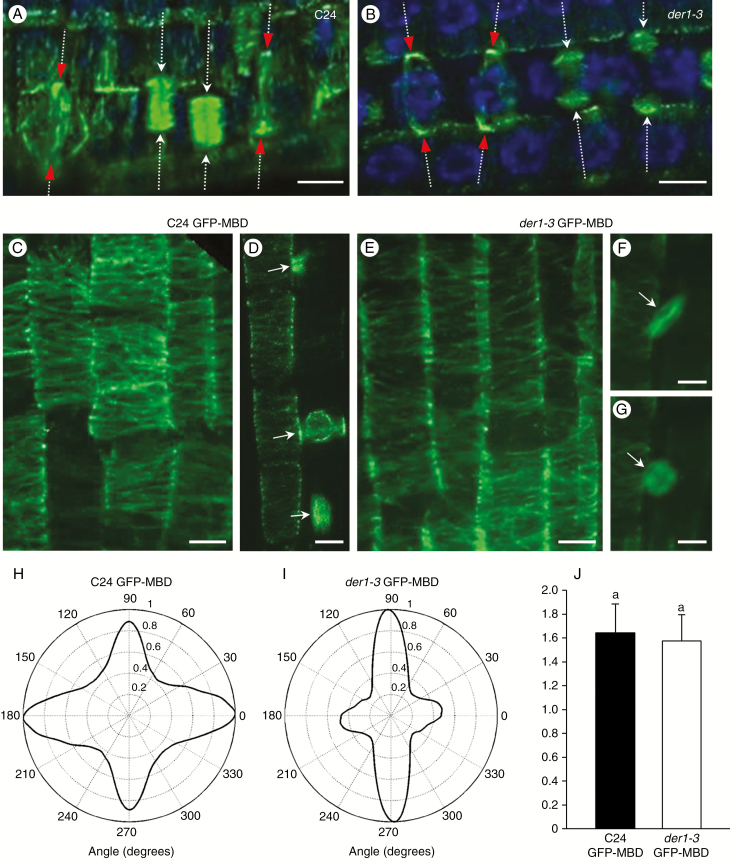
Microtubule organization in root cells of C24 wild-type and *der1-3* mutant plants. (A, B) Microscopic documentation of cortical and mitotic microtubules in root cells of C24 wild-type and *der1-3* mutant after immunofluorescence localization with anti-tubulin antibody. Positions of PPBs in prophase cells are indicated by red arrowheads while positions of phragmoplasts in telophase and during the cytokinesis are indicated by white arrows. All arrows indicate orientations of CDPs. DNA in nuclei is counterstained with DAPI. (C–G) Microscopic documentation of cortical and mitotic microtubules in root cells of living transgenic C24 wild-type and *der1-3* mutant plants harbouring the microtubule fluorescent marker GFP-MBD. Cortical microtubules in root epidermal cells of C24 wild-type (C) and *der1-3* mutant (E) and mitotic microtubules in dividing root epidermal cells of C24 wild-type (D) and *der1-3* mutant (F, G). The same dividing cell in the anaphase stage with a mitotic spindle (F) and in the subsequent telophase with an early phragmoplast (G) is shown in a *der1-3* mutant. Arrows indicate orientation of the PPB in prophase (D), the equatorial plane of the mitotic spindle (D, F) and the midzone of the phragmoplast (D, G). (H, I) Quantitative analysis of cortical microtubule angular distribution in root epidermal cells of C24 wild-type (H, analysed microtubules from C) and in root epidermal cells of *der1-3* mutant (I, analysed microtubules from E). (J) Skewness of cortical microtubules in root epidermal cells of living transgenic C24 wild-type and *der1-3* mutant plants harbouring the microtubule fluorescent marker GFP-MBD. Data are from four or five cells of five or six plants. Scale bars = 5 µm.

Altogether, the results described above indicate that the wavy pattern of root growth in the *der1-3* mutant is most probably caused by deregulation of CDP orientation (Figs 2 and 3) depending on altered organization of the actin cytoskeleton (Fig. 5). To provide a more complex overview of the cytoskeleton-regulating phenotype of the *der1-3* mutant, we also characterized the organization of microtubules. Cortical and mitotic microtubules in root cells of C24 wild-type and the *der1-3* mutant were visualized *in situ* by immunofluorescence localization using an anti-tubulin antibody. In addition, transgenic C24 and *der1-3* mutant plants carrying the microtubule fluorescent marker GFP-MBD were used for live-cell imaging of microtubules in root cells. The CDP is clearly defined by the positioning of the PPB during preprophase and prophase and by the orientation of the phragmoplast during telophase and subsequent cytokinesis. Most cell divisions in the root meristem are proliferative and contribute to the increasing number of successive cells in single cell files, where the CDP is oriented transversely to the main root axis. Thus, we observed regular transverse orientation of the CDP in dividing root cells of the C24 wild-type (Fig. 6A). On the other hand, we observed frequent deviation of the CDP from its expected transverse orientation in root meristematic cells of the *der1-3* mutant (Fig. 6B). Importantly, the organization of cortical (Fig. 6C) and mitotic (Fig. 6D) microtubules in root epidermal cells of control C24 did not differ significantly from that in root epidermal cells of the *der1-3* mutant, as revealed by live-cell imaging (Fig. 6E–G). Determination of the CDP was apparent in dividing cells of control C24, showing coordinated orientation of the PPB, the equatorial plane of the mitotic spindle and the phragmoplast. All of these were transversely oriented to the axis of root cell files (Fig. 6D). On the other hand, the CDP orientation of the dividing cells was not so strictly determined in the *der1-3* mutant as it was frequently observed in oblique orientation with respect to the axis of root cell files (Fig. 6F, G). Because quantitative analysis of cortical microtubule angular distribution in root epidermal cells of control C24 (Fig. 6H) and *der1-3* mutant (Fig. 6I) did not reveal significant differences and no significant differences were found in skewness, the degree of cortical microtubule bundling in root epidermal cells between control C24 and *der1-3* mutant (Fig. 6J), we can conclude that the point mutation of *ACT2* in the *der1-3* mutant does not have a considerable influence on the organization of the microtubule cytoskeleton in root cells.

## DISCUSSION

A single-point mutation in the locus *DER1* (*deformed root hairs1*) leads to the generation of mutant plants impaired in root hair development. The root hair initiation site is not typically positioned on the outer tangential cell wall of the trichoblast and after bulge formation the growth of the root hair tip is abolished. As a result, root hairs of *der1* mutant plants are very short (Ringli *et al.*, 2002). The mutated locus is localized in the sequence of the *ACTIN2* gene, encoding a major vegetative actin in *A. thaliana*. Until now, only the root hair phenotype has been described in *der1* mutants showing no obvious aberrations in plant development (Ringli *et al.*, 2002). The root hair phenotype of the *der1-3* mutant was also confirmed in our study. However, our thorough plant phenotyping and cellular analysis of the cytoskeleton in different cell types revealed that this single-point mutation in the *ACTIN2* gene had a more general influence on plant growth and development.

The first apparent developmental difference was delay in germination of the *der1-3* seeds. During the first 24 h after imbibition only around 50 % of *der1-3* seeds were germinated in comparison with almost 100 % germinating C24 wild-type seeds. Due to this delay in germination, seedlings of *der1-3* mutant were visually smaller, but parameters of average root growth of the *der1-3* mutant were similar to those of control C24 wild-type roots. Similar results were reported for the homozygous *act7-1* mutant, which is defective in the expression of another vegetative actin gene, *ACT7*. Seed germination of the *act7-1* mutant was also about 24 h delayed in comparison with the control wild-type seeds, although without any other changes in growth and further development of germinated seedlings (Gilliland *et al.*, 2003). Slower germination of seeds can significantly influence further seedling development, but it remains to be determined how genetically disturbed organization and dynamics of the actin cytoskeleton are involved in the delay in the seed germination process.

In order to characterize the organization of the actin cytoskeleton, we prepared transgenic C24 and *der1-3* mutant lines carrying GFP-FABD2, a molecular marker for the actin cytoskeleton. Expression of GFP-FABD2 in transgenic plants does not cause detectable adverse effects on plant morphology or development and thus it is generally accepted as a vital marker for the visualization and characterization of the actin cytoskeleton in plants (Voigt *et al.*, 2005; Wang *et al.*, 2008). In general, the actin cytoskeleton in plant cells consists of individual AFs and actin bundles. The network of individual AFs is randomly dispersed in the cortical cytoplasm and, although they are dynamic, they have a shorter lifetime, while actin bundles are mostly oriented longitudinally along the cell’s longer axis; they are less dynamic but have a longer lifetime (Henty-Ridilla *et al.*, 2013). The organization of AFs in cells of the *der1-3* mutant displayed some considerable alterations in comparison with control cells. Individual AFs were shorter in the *der1-3* mutant. More importantly, however, actin bundles were also shorter and thicker than in the control and did not span the entire length of the epidermal cells in cotyledons, hypocotyls and roots. Analysis of actin cytoskeleton organization in living plants harbouring the fluorescent marker GFP-FABD2 was validated by an alternative method of actin labelling in fixed cells using Alexa–phalloidin staining. Both independent methods confirmed the altered configuration of the actin cytoskeleton in different cell types of the *der1-3* mutant. Quantitative determination of actin cytoskeleton orientation demonstrated considerable differences between the *der1-3* mutant and the control C24. Cells of C24 plants contained especially longitudinally oriented actin bundles, while these were mostly obliquely and sometimes transversely oriented in cells of the *der1-3* mutant. Analogous orientations of AFs have been reported in the *act2-1* mutant. Root hairs of the *act2-1* mutant often contained transversely oriented AFs, in contrast to the longitudinally oriented AFs in wild-type plants (Kandasamy *et al.*, 2009). Moreover, similar results have also been published for *act2-2D* and *act2-5* mutants. For example, the *act2-2D* mutant (single-point mutation) had shorter actin bundles in root epidermal cells and showed defects in actin polymerization (Nishimura *et al.*, 2003). The T-DNA insertional mutant *act2-5* showed a modified actin cytoskeleton pattern, where AFs were shorter and unbundled (Lanza *et al.*, 2012). Our data are consistent with these studies and suggest that changes in the organization of the actin cytoskeleton are typical of various mutations in the *ACT2* gene, including *der1-3*. This clearly indicates an important function of ACT2 in the establishment and maintenance of the correct organization of the actin cytoskeleton during plant growth and development.

In general, involvement of ACT2 has been considered mainly in the two major cellular functions, namely root hair formation and cell elongation (e.g. Ringli *et al.*, 2002; Nishimura *et al.*, 2003). Our analysis revealed that, although root growth rate does not differ from the C24 wild-type, roots of the *der1-3* mutant show a wavy growth pattern. Therefore, we documented root growth using different microscopic methods in order to reveal the main reason for root waving. Based on live-cell imaging of transgenic *der1-3* plants harbouring the actin cytoskeleton marker GFP-FABD2, vital labelling of cell membranes using FM4-64 and immunofluorescence whole-mount labelling of microtubules, we confirmed alterations in the cell division pattern in the root meristem. The root meristematic zone of the *der1-3* mutant regularly contained a significantly higher number of shifted cell division planes in comparison with the control C24 plant. The significantly higher number of oblique CDPs deviating from transverse orientation (with respect to the longitudinal root axis) at <90° (therefore not assuming longitudinal orientation) could generate random and asymmetrical forces within the root that lead to a wavy root growth pattern.

However, immunolocalization and live-cell imaging of microtubules revealed that the organization of both cortical and mitotic microtubules in root cells of the *der1-3* mutant does not show any considerable alterations from the normal microtubule organization in root cells of control C24 plants. What was clearly evident, however, was a higher number of shifted CDPs and accordingly tilted mitotic microtubule systems (PPBs, spindles and phragmoplasts) in dividing root meristematic cells of the *der1-3* mutant compared with control C24 plants. The data presented here thus suggest that the actin cytoskeleton plays an important role in the proper determination of the CDP. Microtubules have been reported to associate with AFs during the formation of the PPB already in the prophase stage of cell division (Takeuchi *et al.*, 2016). In subsequent stages of cell division, cortical AFs are less abundant in the future cortical division site, forming the so called actin-depleted zone (Hoshino *et al.*, 2003), but are further involved in the progression of cytokinesis within the expanding phragmoplast. Therefore, the actin cytoskeleton plays relevant roles in cell division and in CDP determination in both asymmetrically and symmetrically dividing cells (Van Damme *et al.*, 2007; Rasmussen *et al.*, 2013; Kojo *et al.*, 2013, 2014). Involvement of the actin cytoskeleton in the determination of CDP orientation has also been tested by application of actin-depolymerizing drugs during preprophase and prophase, leading to misorientation of the CDP in dividing tobacco BY-2 cells (Sano *et al.*, 2005). Finally, defects in CDP orientation were also encountered in plants with reduced expression of *ACT7*, a gene encoding an actin isoform highly expressed during mitosis (Gilliland *et al.*, 2003).

In conclusion, the wavy pattern of root growth, changes in the phenotype of mutant plants and the altered organization of the actin cytoskeleton in different cell types of *der1-3* mutant plants indicate that mutation in the *DER1* gene locus of *ACTIN2* has a more general influence on plant morphology, growth and development than only interference with root hair formation. The changes in mutant plants described here are mediated through alterations of the actin cytoskeleton. Better understanding of the molecular mechanisms of how the actin cytoskeleton contributes to the proper orientation of cell division and what is the role of ACTIN2 isoforms in this process will require more detailed analysis in future studies.
